# Protective effect of 3-O-methyl quercetin and kaempferol from *Semecarpus anacardium* against H_2_O_2_ induced cytotoxicity in lung and liver cells

**DOI:** 10.1186/s12906-016-1354-z

**Published:** 2016-09-29

**Authors:** A. D. Naveen Kumar, Ganesh Babu Bevara, Laxmi Koteswaramma Kaja, Anil Kumar Badana, Rama Rao Malla

**Affiliations:** Department of Biochemistry, Institute of Science, GITAM University, Visakhapatnam, India

**Keywords:** Hydrogen peroxide, Flavonoids, Lung cells, Liver cells

## Abstract

**Background:**

Hydrogen peroxide is continuously generated in living cells through metabolic pathways and serves as a source of reactive oxygen species. Beyond the threshold level, it damages cells and causes several human disorders, including cancer.

**Methods:**

Effect of isolated 3-O-methyl quercetin and kaempferol on H_2_O_2_ induced cytotoxicity, ROS formation, plasma membrane damage, loss of mitochondrial membrane potential, DNA damage was evaluated in normal liver and lung cells. The RT-PCR analysis used to determine Nrf 2 gene expression. Calorimetric ELISA was used to determine Nrf2 and p-38 levels. Expression of SOD and catalase was analyzed by Western blot analysis.

**Results:**

The present study isolated 3-O-methyl quercetin and kaempferol from the stem bark. They protected normal lung and liver cells from H_2_O_2_ induced cytotoxicity, ROS formation, membrane damage and DNA damage. Pre-treatment with 3-O-methyl quercetin and kaempferol caused translocation of Nrf2 from cytosol to nucleus. It also increased expression of p-p38, Nrf2, SOD and catalase in H_2_O_2_ treated lung and liver cells.

**Conclusion:**

The flavonoids isolated from *S. anacardium* significantly reduced H_2_O_2_ induced stress and increased expression of Nrf2, catalase and superoxide dismutase-2 indicating cytoprotective nature of 3-O-methylquercetin and kaempferol.

**Electronic supplementary material:**

The online version of this article (doi:10.1186/s12906-016-1354-z) contains supplementary material, which is available to authorized users.

## Background

Hydrogen peroxide is a physiological constituent of living cells. It is generated by inflammatory and vascular cells and is reported to induce oxidative stress leading to vascular disease and endothelial cell dysfunction. H_2_O_2_ is continuously produced via diverse cellular pathways and serves as a precursor of a wide range of reactive oxygen species. H_2_O_2_ is relatively unreactive oxygen species and causes deleterious effects by inducing lipid peroxidation and DNA damage. H_2_O_2_ is believed to transduce signalling at intracellular steady-state concentration below 1 μM and above cause oxidative stress induced growth arrest and cell death [1].

Antioxidants from natural sources are known to neutralize the H_2_O_2_ and prevent the incidence of disorders. Therefore, the use of antioxidant compounds possibly will help to alleviate oxidative stress mediated diseases [2]. Schwingel et al. [3] highlighted the therapeutic potentials of methylquercetin and kaempferol.

Various parts of *Semecarpus anacardium* are traditionally used for the treatment of rheumatism, cancer and psoriasis [4]. Stem bark, especially used for the treatment of inflammation and cancer in folk medicine. Ethyl acetate extract of the stem bark reported to show strong inhibitory activity on pro-inflammatory enzymes, cyclooxygenase and acetyl cholinesterase [5]. Recently, anti-diabetic and antioxidant activity of ethanolic extract of stem bark was reported [6]. The acetyl cholinesterase (AChE) activity along with antiinflammatory and analgesic effects of stem bark was investigated [7, 8]. Butein, a main chemical constituent of stem bark was isolated [9]. Previously, we reported protective activity of stem bark extract against Fenton reaction induced lipid peroxidation and heat induced hemolysis [10].

The present study was aimed to isolate and characterize antioxidant compounds isolated from methanolic extract of stem bark and to determine their cytoprotective activity against H_2_O_2_ induced cytotoxicity in human normal lung (L132) and liver (L02) cell lines in vitro.

## Methods

### Chemicals and other reagents

FBS, DMEM, Trypsin solution (0.1 %), MTT, DCFH2DA, Rhodamine-123 were purchased from Sigma, USA. Silica gel (mesh size 230–400), Thin layer chromatography plates (Silica gel 60F254), H_2_O_2_, DMSO along with other chemicals and solvents were purchased from Merck (Bangalore, India).

### Source of plant material

The stem bark of *S. anacardium* was collected from the Eastern Ghats of Vizianagaram region, Andhra Pradesh, India. The samples were identified and Authenticated by the faculty of the Department of Botany, Andhra University, Visakhapatnam. The collected specimen was deposited in their herbarium section of the Botany department (DBH), Andhra University, with a voucher specimen number: 21,922.

### Cell lines

The epithelial cells, which line the lungs are the primary targets for reactive oxygen species. The increased oxidative stress in the liver contributes to both onset and progression of hepatic disorders. Hence, human normal lung epithelial (L132) and liver (L02) cell lines were chosen as models for this study. The cell lines were obtained from the National Center for Cell Science (NCCS), Pune, India. The cells were grown to 70 % confluence in DMEM supplemented with 10 % FBS, L-glutamine (2 mM) and penicillin–streptomycin (100 μg/ml) (Sigma, USA) in a humid atmosphere of 5 % CO_2_ at 37 °C.

### Activity guided isolation of antioxidant compounds

Methanolic stem bark extract of *S. anacardium* (35 g) was subjected to silica gel column chromatography. The column was eluted successively with solvents (300 ml) of increasing polarity, i.e. n-hexane, ethyl acetate and methanol in a gradient manner. The fractions with same Rf value and similar color were pooled for each solvent system. The antioxidant activity of each pooled fraction was determined using the DPPH radical scavenging method [10]. The homogeneity of each fraction was examined by thin layer chromatography with solvent systems such as n-hexane, ethyl acetate and methanol and the spots were visualized with ceric sulfate. Further, the purity of pooled fractions were analyzed by RP-HPLC (Waters) equipped with EZ-Chrom, Elite software and C_18_column (Inertsil ODS 3v-150 mm × 4.6 mm, 5 μ) using methanol, water and acetonitrile solvent systems (10:31:59) at a flow rate of 1 ml/min.

### Structural elucidation of isolated antioxidant compounds

The isolated antioxidant compound(s) was analyzed by Agilent 1100 series LC-MSD with electrospray ionization (ESI) and quadrupole mass analyzer using ammonium hydroxide (0.75 M) as buffering reagent. The analysis was done in negative ion mode under the following conditions: flow rate 0.5 ml/min, nebulizer pressure-25 psi, capillary voltage-3 KV, fragmentor voltage-75 V and drying gas temperature- 350 °C. The spectra were scanned over a mass range of m/z (0–1000) and the functional groups were analyzed using Bruker alpha FT-IR instrument (Software opus 6.5). Isolated antioxidant compound (1 g) was mixed with potassium bromide and analyzed using FT-IR spectrophotometer at room temperature in the range of 4000–500 cm^−1^. The number and position of carbon and hydrogen atoms were determined using ^1^H and ^13^C NMR spectra, recorded on Brukers 500 MHz spectrometer using deuterated chloroform (CDCl3). Trimethyl silane (TMS) was used as an internal standard. Chemical shifts were expressed in parts per million (ppm) and coupling constants were indicated in hertz (Hz).

### MTT assay

MTT assay was performed to study the protective effect of the compound against H_2_O_2_ induced cytotoxicity in L132 and L02 cells. Briefly, overnight grown cells (1 × 10^4^/well) were pretreated with isolated 3-O-methyl quercetin and kaempferol (10–500 μg/ml) followed by freshly prepared H_2_O_2_ (100 μM) for 24 h. After treatment, 20 μl of MTT solution (5 mg/ml) was added to each well and incubated for 2 h. Then 100 μl of DMSO was added. H_2_O_2_ treated wells were used as controls. The absorbance was measured at 570 nm using ELISA reader. The cytotoxicity (%) was calculated as follows: O.D of treated wells - O.D of blank wells/O.D of control wells - O.D of blank wells X 100. The percent of cytotoxicity was used to express the viability.

### Estimation of intracellular ROS using DCFH-DA

Estimation of ROS using DCFH-DA is a rapid and sensitive method [11]. After 24 h treatment, the oxidation-sensitive dye DCFH-DA (5 mg/ml) was added to L132 and L02 cells (4.0 × 10^5^cells/well) and incubated for 30 min. The cells were then collected and the intensity of the fluorescence was measured at an excitation wavelength of 485 nm and an emission wavelength of 535 nm using Hidex plate chameleon TM V (Finland). ROS levels were calculated based on the intensity of fluorescence and expressed as percent control. For imaging, overnight grown cells on poly-L-lysine coated chambers were treated as described above. After treatment, cells were stained with DCFH-DA and images were taken using a fluorescence microscope (Olympus, Japan).

### Measurement of mitochondrial membrane potential (MMP)

The protective effect of isolated 3-O-methyl quercetin and kaempferol on H_2_O_2_ induced mitochondrial membrane integrity was determined by measuring the MMP using the fluorescent dye rhodamine 123 [12]. After the treatment, rhodamine 123 (10 μg/ml) was added and incubated at 37 °C for 1 h. Then cells were collected and the fluorescence was determined using a fluorimeter (Hidex plate chameleon™ V, Finland) at an excitation wavelength of 485 nm and an emission wavelength of 535 nm.

### Lactate dehydrogenase (LDH) release assay

The protective effect of 3-O-methyl quercetin and kaempferol on H_2_O_2_ induced cell membrane damage was determined by using LDH-assay (Agappe-11407002) as per the manufacturer’s instructions. After treatment, cells were spun down by centrifugation at 2500 g for 5 min at 4 °C. The supernatant (100 μl) was mixed with a solution containing pyruvate, NADH, Tris buffer and NaCl and total LDH activity was measured as per protocol.

### Alkaline comet assay

Alkaline comet assay [13] was performed to evaluate the protective effect of 3-O-methyl quercetin and kaempferol against H_2_O_2_ induced DNA fragmentation. After treatment, harvested cells (1 × 10^5^) were layered on slide pre-coated with 0.75 % low melting agarose followed by a third layer of 0.75 % low melting agarose. Subsequently, the slide was exposed to lysing solution for 1 h at 4 °C and electrophoresed at 20 V for 20 min. The slide was dipped in neutralization buffer and treated with ethanol for 5 min before staining with 40 μl of ethidium bromide. The images were taken with a fluorescence microscope (Olympus, Japan) and olive tail movement (OTM) was calculated using Image Pro® plus software. The % OTM was calculated as: (head mean) x tail % DNA/100.

### DNA laddering assay

After treatment, cells were centrifuged at 2000 rpm for 5 min at 4 °C and the supernatant was removed. To the pellet, 20 μl of lysis buffer and 10 μl of RNase cocktail was added and incubated for 30 min at 37 °C. Then 10 μl of proteinase K was added and incubated at 50 °C for 90 min. The sample was mixed with 5 μl of 6X DNA loading buffer and loaded into 1.5 % agarose gel containing 0.5 μg/ml ethidium bromide. The sample was electrophoresed at 35 V. DNA ladders were finally visualized by gel doc system and images were documented.

### In silico docking

The crystal structure of human ERK1, JNK1 and p38α proteins were obtained from a protein data bank (http://www.rcsb.org/pdb/home/home.do) and their ID’s were retrieved as 2ZOQ, 3017 and 4E5B. 2D structures of 3-O-methyl quercetin and kaempferol were designed using cheminformatic software (ACD/Chemsketch, version 12, Advanced Chemistry Development, Inc., Toronto, ON, Canada, www.acdlabs.com, 2015), saved as.mol format and converted to 3D structures by hyperchem (.pdb format). Energy minimization of ligand was done using Discovery Studio (DS). Docking studies were performed using genetic algorithm GOLD [14] (v. 3.1; CCDC, Cambridge, UK).

### Caspase 3 activity assay

The activity of caspase-3 was determined using calorimetric assay according to manufacturer’s instructions (Chemicon International Inc., Temecula, CA). The lysates from H_2_O_2_ treated and isolated compound treated cells were transferred to a 96-well plate and treated with the respective peptide substrate and conjugated with *p*-nitroaniline (Ac-DEVD-pNA). After overnight incubation, caspase 3 activity was measured using a microplate reader at 405 nm [15].

### Determination of Nrf2 levels

Calorimetric ELISA in 96-well plate was performed for assaying Nrf2 levels in untreated and treated cell lysates as per manufacturer’s instructions (Cayman chemicals, USA). Cytoplasmic and nuclear fractions were extracted from cell lysate as per manufacturer’s instructions (Thermo Fisher, USA). The samples were added to microplate pre-coated with Nrf2 specific monoclonal antibody and incubated for 2 h. Excess sample was washed out of the plate, Nrf2 detection antibody was added and incubated for 1 h. Then secondary conjugate was added and incubated for 30 min. Excess conjugate was washed out and TMB substrate was added. After incubation at room temperature for 15 min, the enzyme reaction was stopped and absorbance was read at 450 nm.

### Determination of phospho-p38 levels

Calorimetric ELISA in 96 well plate was used for assaying phospho-p38 levels in untreated and treated cell lysates as per manufacturer’s instructions (Thermo Scientific, USA). Cell lysates were added to microplate pre-coated with phospho-p38 specific monoclonal antibody and incubated for 2 h. Excess of sample washed out of the plate and phospho p38 detection antibody was added and incubated for 1 h. Then secondary conjugate was added and incubated for 30 min. Excess conjugate was washed out and TMB substrate was added. After incubation at room temperature for 15 min the enzyme reaction was stopped and absorbance was read at 450 nm.

### Real time-PCR analysis

After treatment of L-132 and L02 cells (1 × 10^7^) total cellular RNA was isolated by Trizol method according to the manufacturer’s instructions (Sigma, USA). Equal amounts of RNA (2 μg) were primed with oligo (dT) primers and reverse-transcribed using a HS-RT PCR kit (Sigma, USA). Amplification of complementary DNA (cDNA) was performed in a total volume of 20 μl of SYBR Green I Master mix (Roche, Germany) containing the following primers. Nrf2: 5′CACGGATGATGCCAGCCAG3′, 3′GCCCGCCCAGAAGTTCA5′ and Myoglobin: 5′GTCTGAGGACTTAAAGAAG3′3′CTCATGATGCCCCTTCT5′, After an initial denaturation at 95 °C for 10 min, 40 PCR cycles were performed using the following conditions: 95 °C, 15 s; 60 °C, 15 s; and 72 °C, 20s. At the end of PCR reactions, samples were subjected to a temperature ramp from 70 °C to 95 °C, (2 °C/s) with continuous fluorescence monitoring. For each PCR product, a single narrow peak was obtained by melting curve analysis of the specific temperature. Each sample for targeted genes (Nrf2) expression was assayed in the duplicates and the ΔCT method was used to quantify expression levels based on normalization to β-2 myoglobin as a reference standard.

### Western-blot analysis

The protein (25 μg) was resolved in 12 % SDS-PAGE and electro-blotted onto polyvinylidene fluoride (PVDF) membrane. The membranes were blocked overnight at 4 °C with 5 % (*v/v*) non-fat dry milk in Phosphate-buffered saline (PBS) and incubated with specific primary antibodies at 1:10,000 dilutions for 1 h, followed by horseradish peroxidase (HRP) conjugated species specific secondary antibodies (Sigma, USA) (1:5000 dilutions) and immune reactivity of the membranes was detected using the enhanced chemi-luminescence peroxidase substrate (Sigma, USA).

### Statistical analysis

Each experiment was carried out at least three times separately and the data were expressed as mean ± SE. Statistical differences between control and target groups for all experiments were determined using Student’s *t*-test. The statistical significance was determined at 5 (*p* < 0.05) level.

## Results and discussion

Our previous study demonstrated the antioxidant activity of methanolic stem bark extracts of *S. anacardium* [10]. Further, the methanolic extract was subjected to silica gel chromatographic separation which resulted in the isolation of four compounds (Additional file 1: Table S1). The main compounds isolated are 3-O-methyl quercetin (S3) (5.77 %) and kaempferol (S4) (3.75 %).

The antioxidant compound, S3 was isolated as yellow color semisolid with ethyl acetate and methanol systems (25:75). The bright yellow color in TLC analysis with ceric sulfate staining indicates the flavonol nature of the compounds [16] (Data not shown). The absorption maxima at 267 and 342.5 nm confirmed the flavonol nature of S3 antioxidant compound [17]. Bright yellow color semisolid antioxidant compound, S4 isolated with the methanol solvent system (100 %). The presence of a flavonoid skeleton in the antioxidant compound S4 was supported by UV maxima at 255.8 and 368 nm [18] (Additional file 2: Figure S1). HPLC profile of S3 and S4 antioxidant compounds showed homogeneity by having a single peak with retention time of 9.45 and 7.05 min compared to S1 and S2 (Data not shown).

IR absorption data on isolated antioxidant compound, S3 was indicative of the hydroxyl group (3425.75 cm^−1^), aromatic functionalities (1463.37 cm^−1^), carbonyl group (1631.81 cm^−1^) and hetero atom bonds (1260 cm^−1^) [19]. The peaks at 3397.50, 2923 and 1712 cm^−1^ of S4 compound refers to presence of aromatic, hydroxyl (−OH) and carbonyl (C = O) groups, respectively [20]. The ESI-MS of S3 compound showed a major molecular ion peak at m/z 315 [M-H^+^] indicated the molecular mass of 316 and theS4 compound showed a major molecular ion peak at m/z 285 [M-H^+^] indicated the molecular mass of 286 [21].

**Fig. 1:**
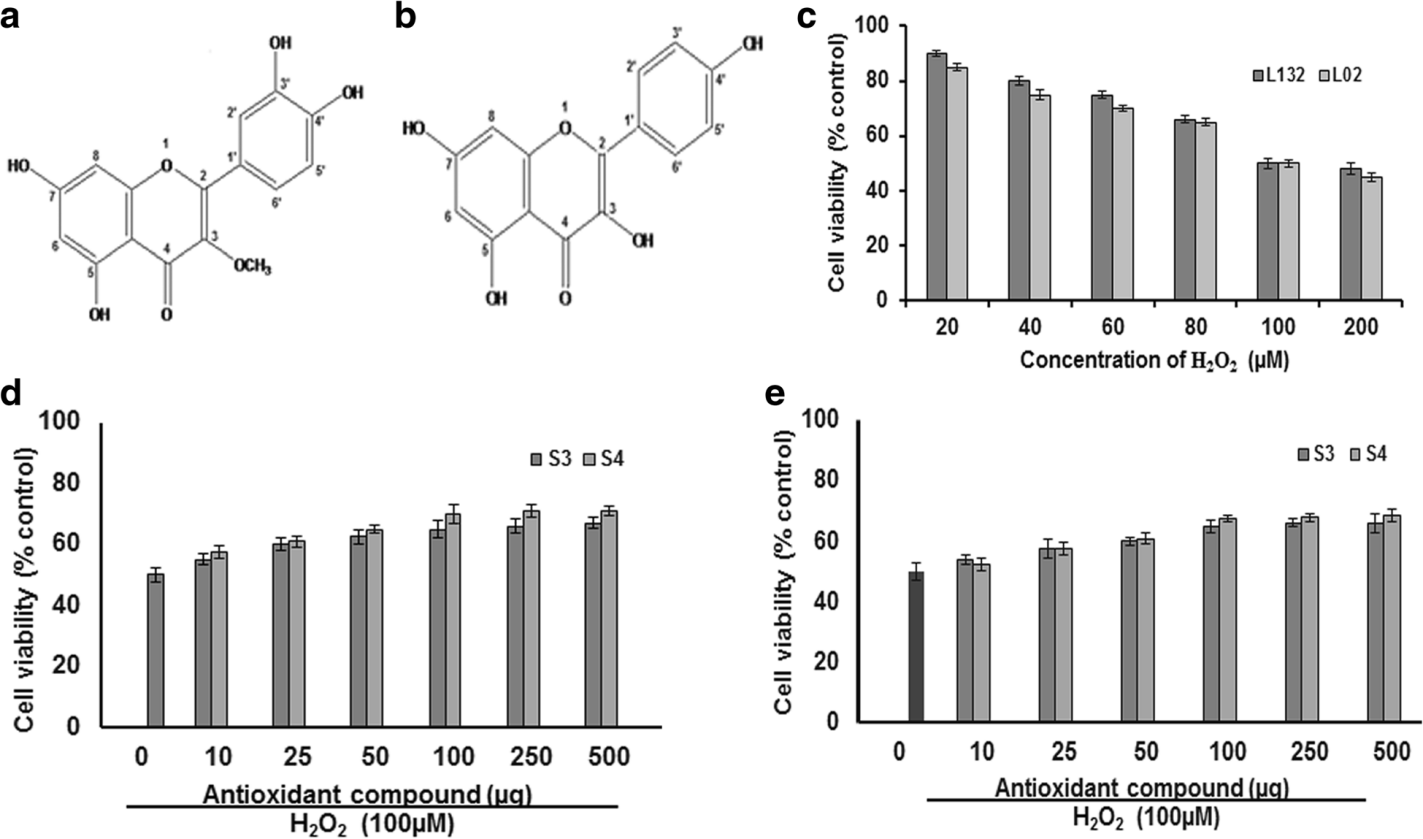
Protective effect of 3-O-methyl quercetin and kaempferol isolated from *S. anacardium* against H_2_O_2_ induced cytotoxicity. Proposed structures of isolated 3-O-methyl quercetin (**a**) and kaempferol (**b**). The Cytotoxic effect of H_2_O_2_ on the viability of the lung (L132) and liver (L02) cells at concentrations ranging from 20 to 200 μM for 24 h and results expressed as percent control (**c**). The Protective effect of isolated 3-O-methyl quercetin (S3) and kaempferol (S4) on H_2_O_2_ induced cytotoxicity in lung (**d**) and liver (**e**) cells. Cells were pretreated with isolated 3-O-methyl quercetin and kaempferol at concentrations ranging from 10 to 500 μg followed by 100 μM H_2_O_2_. The cytotoxic effect was determined by MTT assay and the results were expressed in-terms of viability as percent control. Each value represents mean ± SE of three independent experiments (*n* = 3). The values were significant at *p* < 0.05

The ^1^H NMR spectra of S3 compound showed the signals of singlet and doublet hydrogen atoms at respective positions : 3.73 (3H, s, OCH3-3), 12.72 (1H, s, OH-5), 6.30 (1H, d, J = 1.85 Hz, H-6), 10.68 (1H, s, OH-7), 6.39 (1H, d, J = 2.01 Hz, H-8), 7.59 (1H, d, J = 2.0 Hz, H-2′), 10.74 (1H, s, OH-3′), 10.78 (1H, s, OH-4′), 6.88 (1H, d, J = 8.0 ′Hz, H-5′), 7.41 (1H, dd, J = 1.99, 8.25 Hz, H-6′). From the ^13^C NMR spectra, the δ values of carbon atoms were 152.2 (C-2), 137.4 (C-3), 175.20 (C-4), 159.9 (C-5), 91.5 (C-6), 161.7 (C-7), 96.8 (C-8), 154.3 (C-9), 105.3 (C-10), 123.0 (C-1′), 116.1 (C-2′), 142.9 (C-3′), 58.6 (OCH3-3′), 149.0 (C-4′), 116.1 (C-5′), 119.5 (C-6′). ^1^H and ^13^ C NMR data of these results were consistent with the literature [22]. Thus, based on the above results, we propose the structure of S3 compound proposed as 2-(3,4-dihydroxyphenyl)-5,7-dihydroxy-3-methoxy-4H-chromen-4-one [(3-O-methyl quercetin) (C_16_H_12_O_7_)] (Fig. 1a).

The ^1^H NMR spectra of isolated antioxidant compound S4 showed specific signals for singlet and doublet hydrogen atoms at positions: 6.15 (1H, d, J = 1.4 Hz, H-6), 6.34 (1H, s, H-8), 8.09 (1H, d, J = 8.7 Hz, H-2), 6.81 (1H, d, J = 8.7 Hz, H-3), 6.81 (1H, d, J = 8.7 Hz, H-5), 8.02 (1H, d, J = 8.7 Hz, H-6), 7.89 (2H, d, J = 8.8, H-2′, 6′), 6.91 (2H, d, J = 8.8, H-3′, 5′). The presence of signals in the ^13^C NMR spectra of isolated antioxidant S4 revealed the positions of carbon atoms at: 123.4 (C-1) 150.6 (C-2), 136.8 (C-3), 172.8 (C-4), 157.9 (C-5), 90.5 (C-6), 156.5 (C-7), 96.4 (C-8), 166.4 (C-9), 104.5 (C-10), 128.5 (C-1′), 129.7 (C-2′, 6′), 113.5 (C-3′, 5′), 149.8 (C-4′). The structure was determined by the analysis of ^1^H and ^13^C-NMR data as well as by comparison with previously reported values [23] as [3,5,7-trihroxy-2- (4-hroxyphenyl)-4H-chromen-4-one (kaempferol) (C_15_H_10_O_6_) (Fig. 1b).

**Fig. 2:**
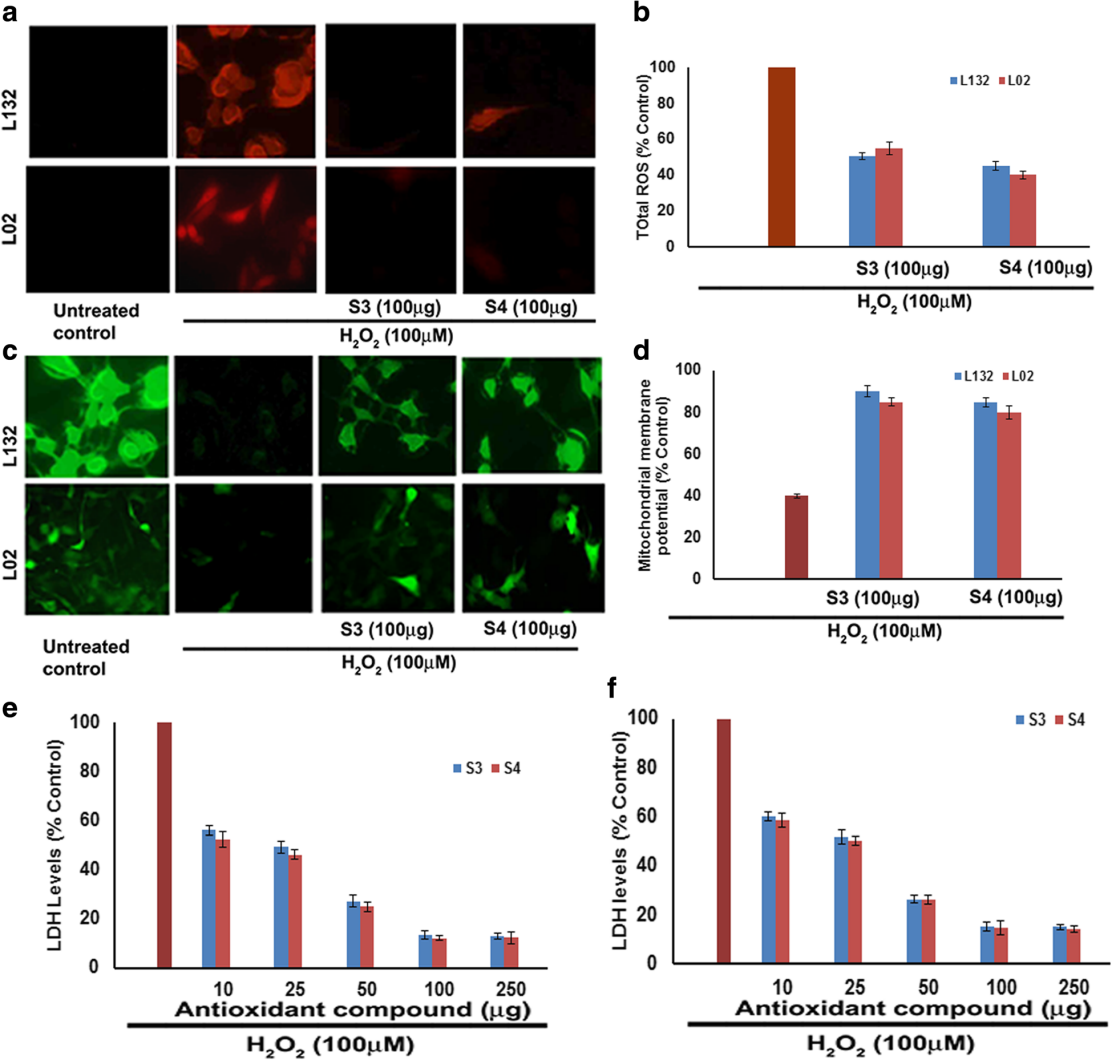
Protective effect of 3-O-methyl quercetin and kaempferol on H_2_O_2_ induced mitochondria and plasma membrane damage in L132 and L02 cells. **a** Cells were pretreated with 3-O-methyl quercetin and kaempferol followed by H_2_O_2_ for 24 h. Cell incubated with 2′,7′-DCFH-DA for 30 min and images were taken using a fluorescence microscope. The intensity of the fluorescence was measured at an excitation wavelength of 485 nm and an emission wavelength of 535 nm and results were expressed in terms of ROS levels as percent control (**b**). Protective effect of 3-O-methyl quercetin and kaempferol on H_2_O_2_ induced mitochondrial membrane potential. After treatment, cells were incubated with Rhodamine 123 (10 μg/ml) for 1 h at 37 °C and change in fluorescence was measured using a fluorimeter (**c**). Densitometric analysis of MMP change in lung and liver cells (**d**). After treatment, total LDH activity in lung (**e**) and liver (**f**) cells was determined as described in materials and methods and results were expressed as percent control. Each value represents mean ± SE of three independent experiments. The values were significant at *p* < 0.05

As a preliminary study, the cytotoxic effect of H_2_O_2_ at a concentration ranging from 20 to 200 μM was evaluated. The results indicate that H_2_O_2_ decreased the cell viability of both lung and liver cells. The decreased cell viability at 24 h treatment may due to H_2_O_2_ induced cytotoxicity. However, 50 % survival of both lung and liver cells was observed with 100 μM H_2_O_2._ Hence, 100 μM H_2_O_2_ used to evaluate the antioxidant activity of isolated compounds (Fig. 1c). Further, the results on the protective effect showed that pre-treatment with both isolated 3-O-methyl quercetin and kaempferol decreased the H_2_O_2_ induced cytotoxicity and a significant protection was observed in both lung and liver cells. The survival rate of lung cells was increased by 10, 20, 25, 30, 32 and 33 % and 15, 22, 30, 40, 42 and 42 % with 10, 25, 50, 100, 250 and 500 μg/ml of isolated 3-O-methyl quercetin and kaempferol, respectively compared to H_2_O_2_ (100 μM) treated cells (Fig. 1d). Similarly, the cell survival rate of H_2_O_2_ treated liver cells increased from by 8, 15, 20, 30, 32 and 32 % and 5, 15, 22, 35, 36 and 37 % with isolated 3-O-methyl quercetin and kaempferol, respectively at a a concentration of 10, 25, 50, 100, 250 and 500 μg/ml compared H_2_O_2_ treated cells (Fig. 1e). The IC_50_ value 3-O-methyl quercetin and kaempferol against lung cells were 25 and 37.5 μg/ml, respectively and liver cells were 30 and 40 μg/ml, respectively. The cytoprotective effect of antioxidants has been partially ascribed in the current research [2]. The protective effect of isolated 3-O-methyl quercetin and kaempferol on H_2_O_2_ induced morphological changes evaluated in both lung and liver cells. Treatment of lung and liver cells with H_2_O_2_ (100 μM) for 24 h caused detachment and shrinkage, which are early symptoms of anchorage dependent cell death. However, pretreatment of cells with isolated 3-O-methyl quercetin and kaempferol prevented detachment and cell shrinkage (Data not shown). Previously, Valko et al., demonstrated the protective effect of natural antioxidants against H_2_O_2_ in cells [24]. To determine the antioxidant propensity of 3-O-methyl quercetin and kaempferol, total ROS levels estimated using dye DCFH-DA. In the presence of ROS, DCFH oxidized to highly fluorescent dichlorofluorescein. The resulting fluorescence used as an index to quantify the ROS levels [25]. The emitted fluorescence was directly proportional to the ROS generated by H_2_O_2_. Treatment of L132 and L02 cells with 100 μM H_2_O_2_ elicited ROS levels, but attenuated significantly with both 3-O-methyl quercetin and kaempferol (Fig. 2a).

The densitometric analysis of DCFH-DA stained cells indicates that pre-treatment of lung (L132) cells with 3-O-methyl quercetin and kaempferol at 100 μg/ml reduced intensity of fluorescence to 50.5 and 45 % respectively, compared to controls. Whereas, treatment of liver (L02) cells with 3-O-methyl quercetin and kaempferol (100 μg/ml) reduced the intensity of fluorescence to 55 and 40 %, respectively (Fig. 2b). The fluorescence intensity decreased by both 3-O-methyl quercetin and kaempferol indicating their potent antioxidant activity.

Free radical generation causes damage and increases permeability of the mitochondrial membrane. This decrease in mitochondrial membrane potential is a biomarker of stress induced apoptotic cell damage [26]. To examine the protective nature of 3-O-methyl quercetin and kaempferol against H_2_O_2_ induced apoptosis, mitochondrial membrane potential (MMP) was measured using rhodamine 123 [27]. The MMP was decreased after treatment with 100 μM H_2_O_2_ compared to untreated control which may be due to the depolarization of mitochondrial membrane. However, treatment of both lung (L132) and liver (L02) cells with 3-O-methyl quercetin and kaempferol at 100 μg/ml prior to treatment with H_2_O_2_, showed a significant recovery of fluorescence intensity indicating the mitochondrial protective nature of isolated antioxidant compounds (Fig. 2c). The densitometric analysis showed that pre-treatment with 3-O-methyl quercetin and kaempferol restored the fluorescence intensity by 50 and 45 %, respectively, in L132 cells and 45 and 40 %, respectively, in L02 cells compared to untreated controls (Fig. 2d). Previously, Eissa et al., reported protective effect of phenolic compounds by attenuating ROS induced damage [28].

LDH is a stable cytoplasmic enzyme present in all cells and is rapidly released into the extracellular environment upon plasma membrane damage [29]. The results of protective effect of 3-O-methyl quercetin and kaempferol showed that H_2_O_2_ induced release of LDH was decreased with 3-O-methyl quercetin and Kaempferol (10 to 250 μg/ml) treatment. The analysis of the results indicates that pre-treatment with 3-O-methyl quercetin decreased the release of LDH by 56.1, 49.2, 27.2, 13.3, and 13 % at 10, 25, 50, 100 and 250 μg/ml, respectively, whereas kaempferol by 52.3, 46.1, 25.0, 12.1 and 12.2 % respectively, in lung cells compared to H_2_O_2_ treated control (Fig. 2e). Further, treatment of liver cells with isolated 3-O-methyl quercetin at 10, 25, 50, 100 and 250 μg/ml decreased the release of LDH by 60.3, 52.0, 26.5, 15.5 and 15.1 %, respectively, whereas kaempferol treatment at the same concentration reduced the LDH by 58.6, 50.4, 26.4, 14.8 and 14.4 % respectively (Fig. 2f). These results signify the dose dependent protective effect of isolated antioxidant compound(s) against H_2_O_2_ induced membrane damage. Similar observations were reported by earlier studies [30].

In recent years, an increased attention is being paid on health and nutritional benefits of medicinal plants. Phenolic compounds are well-known as radical scavengers or radical-chain breakers and they strongly eliminate oxidative free radicals [31]. In biological systems, superoxide radicals converted into hydrogen peroxide in the presence of the superoxide dismutase enzyme. H_2_O_2_ generates hydroxyl radicals in the presence of transition metal ions such as iron and copper leads to DNA strand scission and genomic instability [32].

**Fig. 3:**
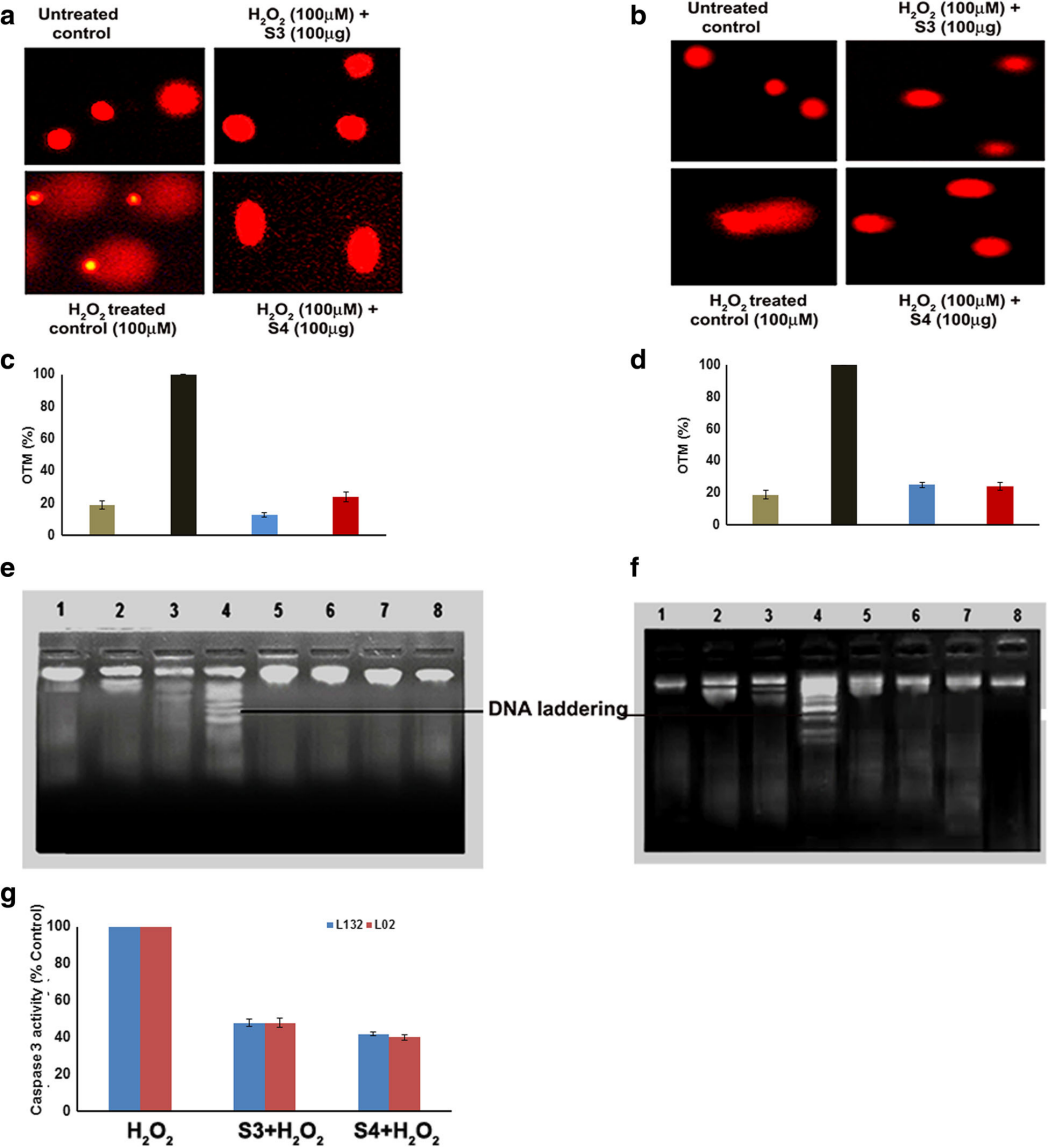
Protective effect of 3-O-methyl quercetin and kaempferol on H_2_O_2_ induced cell death. Lung (**a**) and liver (**b**) cells were pretreated with 100 μg of isolated 3-O-methyl quercetin and kaempferol separately followed by H_2_O_2_ (100 μM) and DNA damage was observed using fluorescence microscopy. Olive tail movement of DNA in lung (**c**) and liver cells (**d**) calculated using image pro plus software. Cells not treated with H_2_O_2_ were served as untreated control and only H_2_O_2_ treated cells were served as a control. Lung (**e**) and liver (**f**) cells pretreated with isolated 3-O-methyl quercetin and kaempferol (100 μg) followed UV or H_2_O_2_ (100 μM) alone or UV and H_2_O_2_ (100 μM). DNA isolated and electrophoresed to observe the band pattern. Lane (1) indicates the DNA of untreated cells, Lane (2) and (3) indicates DNA of UV and H_2_O_2_ treated cells, respectively. Lane (4) indicates the DNA of UV and H_2_O_2_ treated cells. Lane (5) and (6) indicates to the DNA of pre-treated cells with isolated 3-O-methyl quercetin and kaempferol at concentration of 100 μg, respectively H_2_O_2_ Lane (7) and (8) indicates the DNA of cells treated with isolated 3-O-methyl quercetin (S3) and kaempferol (S4) alone at 100 μg concentration. Effect of isolated 3-O-methyl quercetin and kaempferol (100 μg) on caspase 3 activity (**g**). Each value represents mean ± SE of three independent experiments. The values were significant at *p* < 0.05

The comet assay is a relatively simple, but sensitive and well validated method for detecting DNA fragmentation in apoptotic cells [13]. Olive tail moment (OTM) was used as parameter to reflect DNA damage. When lung (L132) and liver (L02) cells were treated with 100 μM H_2_O_2_ alone, caused DNA fragmentation. When lung (L132) cells pretreated with isolated 3-O-methyl quercetin at 100 μg/ml prevented the DNA damage by 87 % and kaempferol by 76 % (Fig. 3a and c). Pre-treatment of Liver cells (L02) with 3-O-methyl quercetin decreased the DNA damage by 75 % and kaempferol by 76 %, compared to H_2_O_2_ treated cells (Fig. 3b and d). The above results clearly demonstrated DNA damage protective role of isolated 3-O-methyl quercetin and kaempferol. Previously, the protective role of flavonols against hydrogen peroxide on DNA damage was reported [33].

This study analyzed the protective role of isolated 3-O-methyl quercetin and kaempferol against both UV and H_2_O_2_ induced damage on genomic DNA of both lungs (L123) and liver (L02) cells. The single band in lane 1 indicates no shearing of DNA during isolation. However, multiple bands in lane 2 and 3 indicated fragmentation of DNA by UV irradiation and 100 μM H_2_O_2,_ respectively. Lane 4 showed the shearing of DNA indicated the formation of DNA laddering mediated by both UV irradiation and 100 μM H_2_O_2._ However, absence of fragmentation of DNA in cells pre-treated with isolated 3-O-methyl quercetin and kaempferol (100 μg/ml) in both lanes 5 and 6 indicated a protective effect of isolate 3-O-methyl quercetin and kaempferol. Lane7 and 8 showed an absence of DNA fragments with isolated 3-O-methyl quercetin and kaempferol alone indicated absence of cytotoxicity nature of 3-O-methyl quercetin and kaempferol, respectively (Fig. 3e and f).

A vast number of studies have demonstrated that H_2_O_2_ treatment induces apoptosis through activation of caspase 3 [34]. In order to assess the protective effect of 3-O-methyl quercetin and kaempferol against H_2_O_2_ induced apoptosis in lung and liver cells, caspase-3 activity was determined. The results show that H_2_O_2_ induced activation of caspase 3 reduced by 3-O-methyl quercetin and kaempferol to 48 and 42 % respectively, in lung cells compared to control. Similarly, H_2_O_2_ mediated activation of caspase 3 reduced by 3-O-methyl quercetin and kaempferol to 48 and 40 % respectively, in liver cells (Fig. 3g). The results suggest that apoptosis caused by H_2_O_2_ prevented by 3-O-Methyl quercetin and kaempferol.

Flavonoids exert therapeutic effects through induction of cytoprotective response, prevention of procarcinogen activation, detoxifying activated carcinogens by enhancing conjugation and excretion [35]. Previous reports have demonstrated that flavonoids interact with proteins of various cellular systems and participate in various signalling cascade events of cancer and ROS mediated disorders [36].

The mitogen-activated protein kinases (MAPKs) are a family of highly related kinases consisting of the extracellular signal-regulated protein kinases (ERKs), the c-jun N-terminal kinases (JNKs), the p38 kinases and other kinases. Studies have reported that, p38 MAPK has been positively related to cell survival [37] and stress induced apoptosis [38]. Further studies have reported that function of p38α depends on the cell type and the stimuli [39].

**Fig. 4:**
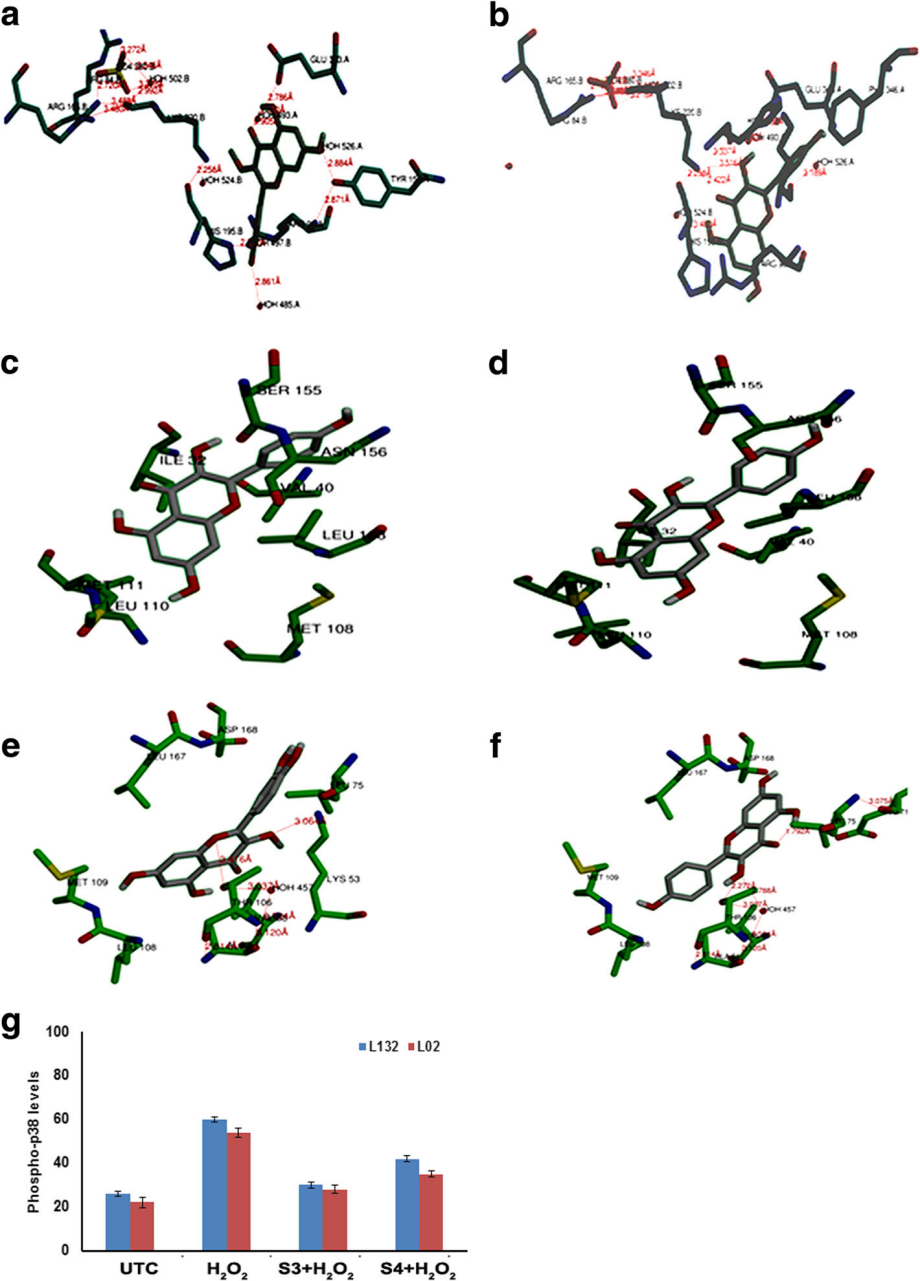
**a**–**f** Docking of isolated 3-O-methyl quercetin and kaempferol with ERK1, JNK1 and p38α. Interactions of 3-O-methyl quercetin with ERK1 (**a**), JNK1 (**b**) and p38α (**c**); kaempferol with ERK1 (**d**), JNK1 (**e**) and p38α (**f**). The side chains of amino acid residues shown as sticks and subsites indicated. Pictures for representation were generated from GOLD DOCK studies. **g** Effect of 3-O-methyl quercetin and kaempferol on expression of phospho-p38. Cells pre-treated with isolated 3-O-methyl quercetin and kaempferol at concentration 100 μg followed by 100 μM H_2_O_2_ and Phospho-p38 levels determined and results were plotted (**g**)

In the present study, GOLD suite used to determine the binding interaction between the binding sites of target proteins ERK1, JNK1 and p38α with 3-O-methyl quercetin and kaempferol (Fig. 4a–f). The Gold scores of ERK1 with 3-O-methyl quercetin and kaempferol were 32.96 and 36.56, respectively, JNK1 was 33.90 and 29.02, respectively and p38α was 45.70 and 43.75, respectively. These results indicate that both 3-O-methyl quercetin and kaempferol had highest docking scores towards p38α, which refers to the high interacting ability of these antioxidant compounds with p38α.

Further, the effect of 3-O-methyl quercetin and kaempferol on H_2_O_2_ induced phosphorylation of p38, ERK and JNK were tested. The results indicated that phospho-p38 levels were significantly increased in H_2_O_2_ treated lung and liver cells by 2.3 and 2.45 folds compared to untreated control. However, 3-O-methyl quercetin and kaempferol at concentration of 100 μg/ml attenuated the expressions by 50.3 and 48.5 %, respectively in lung cells and 45.7 and 42.6 %, respectively in liver cells (Fig. 4g). Activated p38 controls cell proliferation, differentiation and apoptosis [40]. 3-O-methyl quercetin and kaempferol treatment did not show any change in pERK and pJNK levels (Data not shown).

In mammals, intracellular redox homeostasis maintained mainly through the transcriptional control of an array of antioxidative genes. Naidu et al., reported that Nrf2 activation could be attenuated by the over expression of p38-MAPKs [41]. It has also been described that induction of the antioxidant responsive element (ARE) through Nrf2 [42].

NF-E2-related factor 2 (Nrf2) is a CNC-bZIP transcription factor, which regulates the basal and inducible expression of a wide array of antioxidant genes. Following dissociation from the cytosolic protein Keap1, Nrf2 rapidly translocate and accumulates in the nucleus and transactivate the antioxidant response element in the promoter region of many antioxidant genes. The MAPK signalling system responds to diverse stimuli, including oxidative stress and is implicated in the Nrf2 induction [43]. Nrf2 seems to play an important role in the protection against H_2_O_2_ induced liver injury [44]. Thus, Nrf2 regulates the response to cellular stress and cell survival [45].

**Fig. 5:**
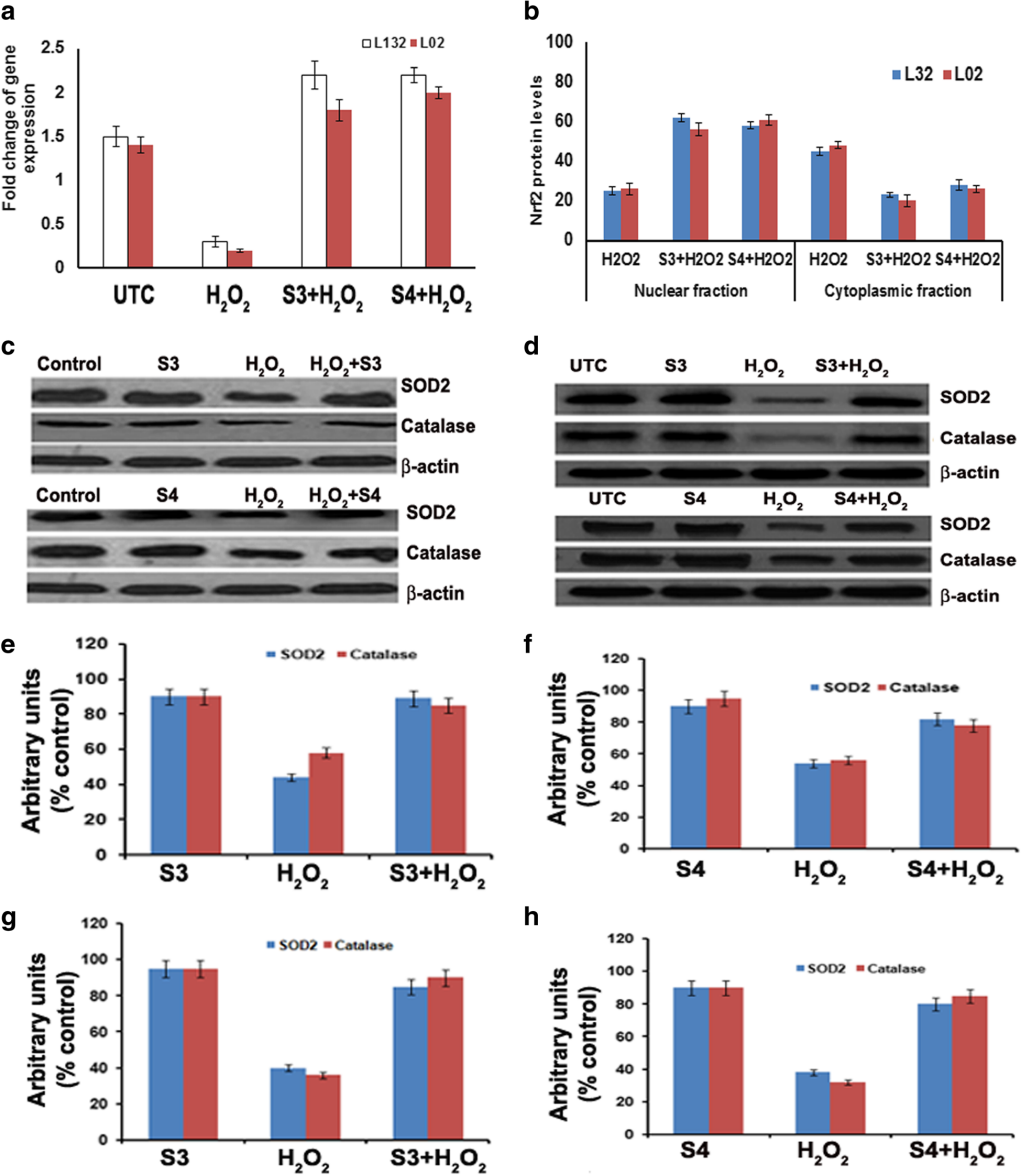
Effect of isolated 3-O-methyl quercetin (S3) and kaempferol (S4) on expression ofoxidative stress markers. **a** Fold change of Nrf-2 gene expression in lung and liver cells pre-treated with 3-O-methyl quercetin and kaempferol. The Nrf2 levels in nuclear and cytoplasmic fractions of lung and liver cells pre-treated with 3-O-methyl quercetin and kaempferol and results were expressed as percent control (**b**). Western blot analysis of SOD2 and catalase in lung (**c**) and liver (**d**) cells pretreated with 3-O-methyl quercetin and kaempferol (100 μg/ml) followed H_2_O_2_ (100 μM). Quantification of SOD2 and catalase expression in S3 (**e**) and S4 (**f**) treated lung cells and S3 (**g**) and S4 (**h**) treated liver cells by densitometry

The results of gene expression studies shows that pre-treatment of lung and liver cells with 3-O-methyl quercetin and kaempferol (100 μg/ml), increased Nrf2 mRNA expression in H_2_O_2_ treated lung and liver cells (Fig. 5a). This study also observed that Nrf2 levels were more in the cytosolic fraction of H_2_O_2_ treated cells compared to nuclear fraction. However Nrf2 levels were more in nuclear fraction compared to the cytosolic fraction of lung and liver cells treated with 3-O-methyl quercetin and kaempferol prior to treatment with H_2_O_2_ (Fig. 5b). These results indicate that pre-treatment causes translocation of Nrf2 from cytosol to nucleus. Previous study [46] demonstrated that expression of Nrf2 increased by polyphenols. In line with our results, quercetin and kaempferol derivatives have reported to up regulate Nrf2 expression [47]. The apparent correlation between p38 and Nrf2 suggests that Nrf2 could be a downstream target of p38α during the exposure of lung and liver cells to H_2_O_2_ (100 μM). Jang and Surh [48] reported that treatment of PC12 cells with Resveratrol protected against H_2_O_2_ induced cell death, through MAP kinase mediated Nrf2 activation. Thus, this study demonstrated that p38-MAP Kinase regulated pathway might be involved in cellular defense through modulation of Nrf2 and antioxidant enzyme levels.

For further study, the cytoprotective effect of 3-O-methyl quercetin and kaempferol on H_2_O_2_ treated lung and liver cells, expression of antioxidant enzymes such as glutathione peroxidase, superoxide dismutase 2 (SOD2) and catalase (CAT) were determined by western blot analysis. Treatment with 100 μg/ml of isolated antioxidant compounds did not exhibit any significant change in SOD2 and CAT expression compared with untreated cells. Cells treated with 100 μM H_2_O_2_ alone resulted in the decreased expression of CAT and SOD2 in both cells. However, no change in glutathione peroxidase expression with pre-treatment in H_2_O_2_ treated lung and liver cells (Additional file 3: Figure S2). However, pre-treatment with 100 μg/ml of 3-O-methyl quercetin and kaempferol resulted in a significant increase in CAT and SOD2 expression in both cells (Fig. 5c and d).

Densitometric analysis of western blot bands of lung cells reveals that H_2_O_2_ treatment decreased SOD2 and CAT levels by 60 and 40 %, respectively, compared to untreated control, whereas in liver cells by 45 and 42 %, respectively. However, pre-treatment of lung cells with 3-O-methyl quercetin increased SOD2 and CAT levels by 50 and 30 %, respectively, and kaempferol by 25 and 18 %, respectively (Fig. 5e and f). Further, pre-treatment of liver cells with 3-O-methyl quercetin increased the SOD2 and CAT levels by 25 and 28 %, respectively, and kaempferol by 19.5 and 30.5 %, respectively (Fig. 5g and h). Recently, Emamgholipour et al. (2016) [49] reported that peripheral blood mononuclear cells (PBMNCS) showed decreased expression of SOD levels with H_2_O_2_ treatment compared to untreated cells both mRNA expression and activity. Pre-treatment of PBMCS with melatonin, a known antioxidant prior to exposure to H_2_O_2_ caused a significant increase in SOD levels in comparison with PBMNCS treated only with H_2_O_2_ [49]. Interestingly, cells pretreated with 100 μg of isolated antioxidant compounds following exposure to H_2_O_2_ (100 μM) showed a significant increase in levels of CAT and SOD2 compared to H_2_O_2_ treated cells, suggesting cytoprotective nature of isolated antioxidant compounds, 3-O-methyl quercetin and kaempferol in mediating the cell survival.

## Conclusion

The present study isolated 3-O-methyl quercetin and kaempferol from the stem bark. They protected normal lung and liver cells from H_2_O_2_ induced cytotoxicity, ROS formation, membrane damage and DNA damage. Pre-treatment with 3-O-methyl quercetin and kaempferol caused translocation of Nrf2 from cytosol to nucleus. It also increased expression of p-p38, Nrf2, SOD and catalase in H_2_O_2_ treated lung and liver cells. The flavonoids isolated from *S. anacardium* significantly reduced H_2_O_2_ induced stress and increased expression of Nrf2, catalase and superoxide dismutase-2 indicating cytoprotective nature of 3-O-methylquercetin and kaempferol.

## Additional files

Additional file 1: Table S1. Silica gel column chromatography of methanolic extract of *S. anacardium*. (DOCX 52 kb) Additional file 2: Figure S1. UV-vis spectrophotometric analysis of isolated antioxidant compounds. (DOCX 66 kb) Additional file 3: Figure S2. Effect of 3-O-methyl quercetin (a) and kaempferol (b) on expression of Glutathione peroxidase. (JPG 218 kb)

## Abbreviations

CAT: Catalase
DCFH2DA: 2′, 7′-dicholoro-dihydro-fluorescein diacetate
DMEM: Dulbecco’s modified eagle medium
DMSO: Dimethyl sulfoxide
DPPH: 2,2-diphenyl-1-picrylhydrazyl
ERK1: Extracellular signal–regulated kinase 1
FBS: Fetal bovine serum
H_2_O_2_: Hydrogen peroxide
JNK1: c-Jun N-terminal kinase 1
L02: Human normal liver cells
L132: Human normal lung cells
MTT: 3-(4,5-dimethylthiazol-2-yl) -2,5-diphenyltetrazolium bromide
Nrf2: NF-E2-related factor 2
SOD2: Superoxide dismutase 2
