# Supplementary material for: Protective effect of 3-O-methyl quercetin and kaempferol from Semecarpus anacardium against H2O2 induced cytotoxicity in lung and liver cells
Source: BMC Complement Altern Med. 2016 Sep 29;16:376. doi: 10.1186/s12906-016-1354-z (PMC5041319; doi:10.1186/s12906-016-1354-z)
Supplement: Additional file 1: Table S1. — Silica gel column chromatography of methanolic extract of S. anacardium. (DOCX 52 kb) [file 12906_2016_1354_MOESM1_ESM.docx]

**Supplemental Table-S1: Silica gel column chromatography of methanolic extract of**

***S .anacardium*.**

|  | **Fraction No** | **Ratio of solvents** | **Volume**  **(ml)** | **Single spot Pooled fractions** | **Rf Val**  **ue** | **DPPH radical ScavengingActivity**  **(%)** | **Yied**  **(mg)** |
| --- | --- | --- | --- | --- | --- | --- | --- |
| 1  2  3  4  5  6  7.    8  9 | 1-35  36-86  87-150  151-225  226-310  311-375  376-428  429-490  491-540 | n-hexane(100)  n-hexane:Ethyl  acetate (75:25)  n-hexane:Ethyl  acetate (50:50)  n-hexane:Ethyl  acetate (25:75)  Ethylacetate (100)  Ethylacetate: Methanol (25:75)  Ethylacetate: Methanol (50:50)  Ethylacetate: Methanol (75:25)  Methanol (100) | 300  300  300  300  300  300  300  300  300 | 1F1(13-25)  2F1(40-54)  2F2(63-82)  3F1(90-112)  3F2(118-132)  3F1(138-150)  4F1(174-206)  4F2(212-221)  5F1(258-278)  5F2(289-305)  6F1(318-340)  6F2(345-369)  7F1(380-386)  7F2(392-405)  7F1(411-426)  8F1(445-470)  8F2(478-440)  9F1(495-515)  9F2(519-525) | 0.58  0.61  0.60  0.43  0.47  0.35  0.52  0.55  0.38  0.45  **0.82**  0.76  0.48  0.55  **0.79**  **0.88**  0.60  **0.85**  0.59 | 28.54  53.5  52.17  67.45  61.65  55.81  42.05  41.98  62.09  59.25  **90.18 (S1)**  78.2  81.8  62.3  **90.52 (S2)**  **92.7 (S3)**  78.87  **91.59 (S4)**  54.25 | 45  121  258  295  321  276  560  385  335  489  **1610**  456  936  190  **1590**  **2090**  670  **1875**  254 |

**Supplementary Figure-S1: UV-Vis spectrophotometric analysis of isolated antioxidant compounds**

**a)**


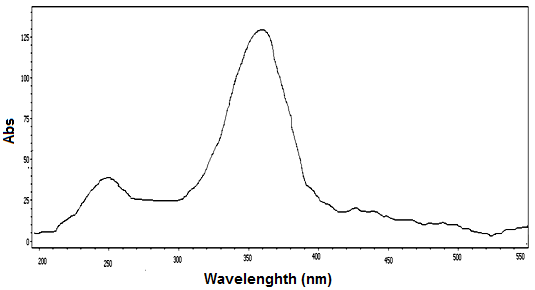


**b)**

**
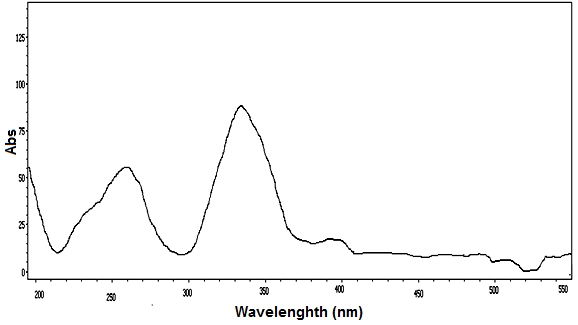
**

UV absorption spectra of isolated antioxidant compounds S3 (a) and S4 (b) was measured in the range of 200 to 550 with an interval of 50 nm.
