# Supplementary material for: Protective effect of 3-O-methyl quercetin and kaempferol from Semecarpus anacardium against H2O2 induced cytotoxicity in lung and liver cells
Source: BMC Complement Altern Med. 2016 Sep 29;16:376. doi: 10.1186/s12906-016-1354-z (PMC5041319; doi:10.1186/s12906-016-1354-z)
Supplement: Additional file 2: Figure S1. — UV-vis spectrophotometric analysis of isolated antioxidant compounds. (DOCX 66 kb) [file 12906_2016_1354_MOESM2_ESM.docx]

a)

b)

**HPLC profile of 3-O- methyl quercetin (a) and kaempferol (b)**
