# Supplementary figures and images for: Protective effect of 3-O-methyl quercetin and kaempferol from Semecarpus anacardium against H2O2 induced cytotoxicity in lung and liver cells
Source: BMC Complement Altern Med. 2016 Sep 29;16:376. doi: 10.1186/s12906-016-1354-z (PMC5041319; doi:10.1186/s12906-016-1354-z)

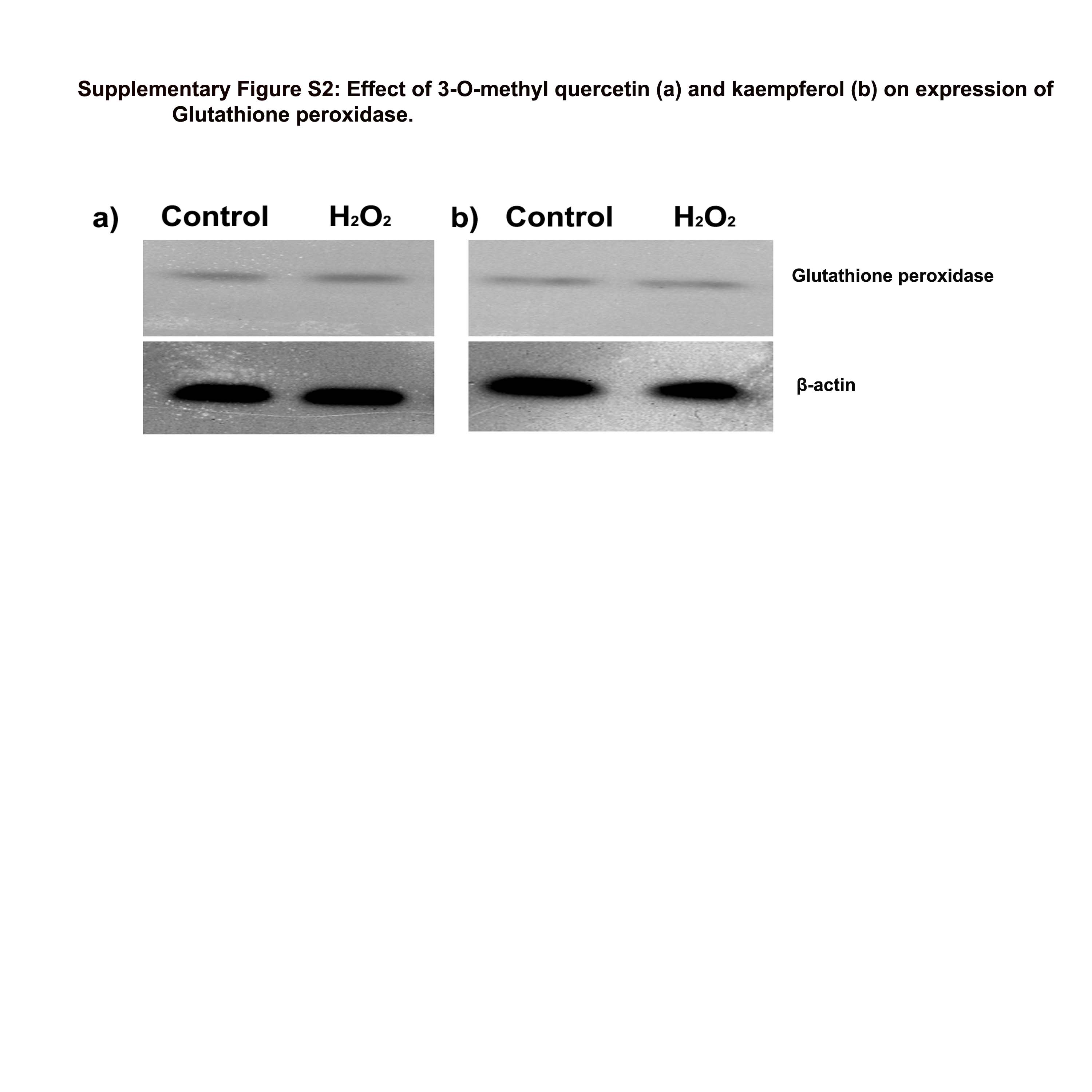

Supplement: Additional file 3: Figure S2. — Effect of 3-O-methyl quercetin (a) and kaempferol (b) on expression of Glutathione peroxidase. (JPG 218 kb) [file 12906_2016_1354_MOESM3_ESM.jpg]
